# Intervention heroes of Mozambique from 1997 to 2015: estimates of maternal and child lives saved using the Lives Saved Tool

**DOI:** 10.7189/jogh.08.021202

**Published:** 2018-12

**Authors:** Ivalda Macicame, Amílcar Magaço, Marta Cassocera, Celeste Amado, Américo Feriano, Sérgio Chicumbe, Jorge Jone, Quinhas Fernandes, Kátia Ngale, Emilia Vignola, Caroline De Schacht, Timothy Roberton

**Affiliations:** 1 Instituto Nacional de Saúde, Ministry of Health, Maputo, Mozambique; 2 Direcçăo Nacional de Saúde Pública, Ministry of Health, Maputo, Mozambique; 3 Johns Hopkins University – Bloomberg School of Public Health, Baltimore, Maryland, USA; 4 Health Alliance International, Maputo, Mozambique

## Abstract

**Background:**

As one of several countries that pledged to achieve the Millennium Development Goals (MDGs), Mozambique sought to reduce child, neonatal, and maternal mortality by two thirds by 2015. This study examines the impact of Mozambique’s efforts between 1997 and 2015, highlighting the increases in intervention coverage that contributed to saving the most lives.

**Methods:**

A retrospective analysis of available household survey data was conducted using the Lives Saved Tool (LiST). Baseline mortality rates, cause-of-death distributions, and coverage of child, neonatal, and maternal interventions were entered as inputs. Changes in mortality rates, causes of death, and additional lives saved were calculated as results. Due to limited coverage data for the year 2015, we reported most results for the period 1997-2011. For 2011-2015 we reported additional lives saved for a subset of interventions. All analyses were performed at national and provincial level.

**Results:**

Our modelled estimates show that increases in intervention coverage from 1997 to 2011 saved an additional 422 282 child lives (0-59 months), 85 450 neonatal lives (0-1 month), and 6528 maternal lives beyond those already being saved at baseline coverage levels in 1997. Malaria remained the leading cause of child mortality from 1997 to 2011; prematurity, asphyxia, and sepsis remained the leading causes of neonatal mortality; and hemorrhage remained the leading cause of maternal mortality. Interventions to reduce acute malnutrition and promote artemisinin-based combination therapy (ACT) for malaria were responsible for the largest number of additional child lives saved in the 1997-2011 period. Increases in coverage of delivery management were responsible for most additional newborn and maternal lives saved in both periods in Mozambique.

**Conclusion:**

Mozambique has made impressive gains in reducing child mortality since 1997. Additional effort is needed to further reduce maternal and neonatal mortality in all provinces. More lives can be saved by continuing to increase coverage of existing health interventions and exploring new ways to reach underserved populations.

Like many sub-Saharan African countries, Mozambique made a commitment to achieve Millennium Development Goals (MDGs) 4 and 5: to reduce its under-five mortality rate (U5MR) by two thirds and its maternal mortality ratio (MMR) by three quarters by 2015 [1]. Results from Demographic and Health Surveys (DHS) in 1997, 2003, and 2011 suggest that Mozambique went a long way to achieving the U5MR target, but not the MMR target. The U5MR decreased from 201 in 1997 to 153 in 2003 and 97 in 2011 [2-4]. The MMR declined from 692 in 1997 to 408 in 2003, but then remained stagnant at 408 in 2011 [2-4].

In the coming years, the Mozambique Ministry of Health aims to “accelerate progress in reducing maternal and neonatal mortality” to achieve Sustainable Development Goals (SDGs) 3.1 and 3.2, as outlined in its Health Sector Strategic Framework (PESS) for 2014-2019 [5]. To successfully prioritize the scale-up of interventions during this next period, it will help to first explore which interventions were most impactful during the previous period. Understanding how the scale-up of interventions has contributed to saving lives in the past will help to set effective targets and expectations going forward.

The Lives Saved Tool (LiST) is a modelling program within the Spectrum software package that has been used in various settings to model the impact of coverage changes on child and maternal mortality [6,7]. Users enter custom input values for baseline mortality rates, cause-of-death distributions, disease incidence, and coverage rates. Outputs can be generated showing how increases in coverage prevent additional deaths over time, affecting mortality rates and causes of death [8]. The number of additional lives saved can be attributed to specific interventions, allowing users to see which interventions contributed the most to reducing deaths and improving population health.

As part of work undertaken with the National Evaluation Platform (NEP) in Mozambique, we conducted a retrospective LiST analysis to estimate the additional lives saved by changes in intervention coverage from 1997 to 2015. The NEP supports effective policy-making for child and maternal health, by conducting secondary analyses of national and provincial data and supplying policy-makers with evidence to inform decisions [9]. Our goal was to highlight the programmatic efforts in Mozambique that were most impactful in reducing child, neonatal, and maternal deaths from 1997 to 2015, so as to inform future efforts to achieve the SDGs and continue to improve the health of women and children throughout the country.

## METHODS

### Study design and data sources

In this retrospective secondary analysis, we modelled national and provincial changes in coverage of child and maternal health interventions between 1997 and 2015, and estimated the impact of those coverage changes on child, neonatal, and maternal mortality in Mozambique. Intervention coverage data were obtained from Demographic Health Surveys (DHS) in 1997, 2003, and 2011; a Multiple Indicator Cluster Survey (MICS) in 2008; an AIDS Indicator Survey (AIS) in 2009; and an Immunization, Malaria and HIV/AIDS Indicators Survey (IMASIDA) in 2015. These surveys represent all of the available household surveys with data on coverage of child and maternal health interventions in the past 20 years that are representative at national and provincial levels.

For national baseline child and neonatal mortality rates we used estimates from the UN Inter-agency Group for Child Mortality Estimation (UN IGME) [10]. For baseline maternal mortality rates we used published estimates from WHO, UNICEF, UNFPA, World Bank Group, and the UN Population Division [11]. We calculated provincial baseline mortality rates using national-to-subnational LiST projections to estimate provincial mortality rates based on differences in provincial coverage to national coverage.

The data sources for baseline cause-of-death estimates for Mozambique at national level were those used by LiST by default and come from WHO mortality estimates [12,13]. For provincial cause-of-death estimates, we created national-to-subnational LiST projections (as above) to estimate provincial cause-of-death estimates based on differences in provincial coverage to national coverage.

### Data analysis

Of the 72 indicators with known effectiveness values that are customizable within LiST, we identified 55 indicators available in the Mozambique household survey data sets, including preventative and curative interventions during pregnancy, childbirth, the neonatal period, and childhood. We used raw survey data to recalculate certain indicators using the software STATA version 13 (Stata Corp, College Station, TX: StataCorp LP), so as to standardize indicators for comparison over time, and to match the indicator definitions expected by LiST. Additionally, we calculated proportions of children stunted and wasted by age category and Z-score, to accurately calculate the impact of interventions on children in different risk categories.

We undertook our analysis with LiST version 5.441 (Spectrum). We manually inputted survey coverage data for the years that were available, and linearly interpolated between these years. For indicators that were not available in survey data sets, or for which only one year of data was available, we flatlined coverage from 1997 to 2015 and these interventions thus had no effect on the model. For indicators where data was available for only two years, or was not available for either 1997 or 2015, we linearly interpolated coverage between the available years, and duplicated (flatlined) coverage before and after the available years.

Once the projections were complete, we used LiST to generate three types of outputs at national and provincial levels: mortality rates, number of deaths by cause, and number of additional lives saved by intervention. For each of these outputs we separated results by age category: neonatal (0-1 month), child (0-59 months), and maternal. Once we had calculated the additional lives saved for each intervention, we identified the 10 interventions that contributed the largest number of additional lives saved nationally, and listed the number and proportion of additional lives saved from the same interventions by province.

While we had data for most indicators for the years 1997, 2003, 2008, 2009, and 2011, we only had data for a limited number of indicators from the 2015 IMASIDA survey. Given that LiST relies on coverage data to calculate accurate results, the limited availability of 2015 data meant that LiST would underestimate the number of lives saved in the period 2011-2015. We therefore chose to present our findings in two time periods: 1997-2011 and 2011-2015. For 1997-2011 we reported all available results. For 2011-2015 we only reported additional lives saved for a subset of interventions.

## RESULTS

### Changes in coverage of interventions

The coverage values for child, neonatal, and maternal interventions that we used as inputs for our LiST projections are available in **Table 1** (national coverage values for single-value indicators) and Appendices S1 & S2 in **Online Supplementary Document(Online Supplementary Document)** (breastfeeding, stunting, and wasting by age category; and provincial coverage values). While there were variations in coverage rates over time and across provinces, most interventions showed a general increase in coverage from 1997 onwards. Coverage of vaccines increased in all provinces of Mozambique from 1997 to 2011, and continued to increase in most provinces until 2015. Coverage of diphtheria-tetanus-pertussis vaccine 3 doses (DPT3) decreased in Tete between 2011 and 2015, and coverage of measles vaccine decreased in Nampula from 2011 to 2015. Coverage of oral rehydration solution (ORS) for diarrhea increased nationally from 1997 to 2011, but decreased in many provinces from 2011 to 2015. Coverage of pneumonia interventions increased steadily from 1997 to 2015. Coverage of malaria interventions increased nationally, although Maputo Province and Maputo City had low coverage of antimalarials in all years. Data from 1997 to 2015 showed consistent increases in coverage of maternal and neonatal interventions in 10 of the 11 provinces, with Manica showing decreasing coverage of antenatal care (ANC4), facility delivery, and skilled birth attendance from 2011 to 2015. All maternal interventions had higher coverage in the provinces from the southern region of Mozambique.

**Table 1:** Intervention coverages at national level from 1997 to 2015

		DHS	DHS	MICS	AIS	DHS	IMASIDA
**1997**	**2003**	**2008**	**2009**	**2011**	**2015**
Preventive	ITN/IRS		5.80%	26.17%		51.45%	
	Improved drinking water source	70.15%	83.15%	83.23%	89.00%	84.00%	
	Water channeled into the household	4.85%	5.26%	6.85%	6.01%	10.82%	
	Improved Sanitary Infrastructures	28.59%	39.40%	41.04%	41.74%	49.09%	
Pregnancy	Antenatalcare (ANC) 4 or more visits	40.77%	52.95%			48.44%	54.60%
	Tetanus vaccine: 2+ doses during the most recent pregnancy	30.94%	58.68%	66.75%		66.26%	
	Intermittent and preventive treatment for malaria during pregnancy			44.10%	40.74%	20.35%	34.20%
Delivery	Skilled birth attendant (births in 2 years before survey)	44.60%	49.89%	55.25%		55.99%	73.00%
	Facility delivery	44.33%	50.91%	58.09%		58.86%	70.30%
Preventive	Vitamin A in the last 6 months (6-59 months of age)		52.00%	71.99%		75.19%	
	Safe excretion of the child's stool		57.49%	56.93%		77.84%	
Vaccine	Received 3 doses of DPT	60.27%	72.73%	70.43%		77.02%	81.60%
	Received measles vaccination	57.78%	76.83%	65.47%		81.58%	82.70%
Curative	ORS for diarrhea	41.87%	48.65%	38.24%		55.08%	45.90%
	Antibiotics for diarrhea					27.69%	
	Search for pneumonia care	38.54%	55.36%	59.07%		53.64%	56.50%
	Antimalarials – Artemisinin compounds for malaria		6.20%	21.72%		22.52%	35.60%

ITN/IRS – Insecticide-treated nets/indoor residual spraying, ORS – oral rehydrationn salts, DPT – diphtheria, pertussis and tetanus

*Disaggregated coverage values for breastfeeding, stunting, and wasting by age category are shown in Appendices S1 & S2 in **Online Supplementary Document(Online Supplementary Document)**. Provincial-level coverage values are also shown in Appendices S1 & S2 in **Online Supplementary Document(Online Supplementary Document)**.

### Changes in mortality

LiST estimates trends in mortality rates arising from changes in intervention coverage. **Table 2** shows the mortality rates estimated by our projections. The national under-five mortality rate (U5MR) decreased from 196 in 1997 to 145 in 2011. The provinces in the northern region (Niassa, Cabo Delgado, Nampula) showed the greatest percentage reduction, while Maputo Province showed the smallest percentage reduction. The neonatal mortality rate (NMR) dropped from 50 in 1997 to 41 in 2011. Cabo Delgado was among the provinces with a high NMR in 1997 and its percentage reduction was only 7%. Maputo Province already had a low NMR in 1997, but showed one of the greatest reductions in mortality from 1997 to 2011. Gaza and Maputo City showed slight increases in NMR from 1997 to 2011. The national maternal mortality ratio (MMR) decreased from 870 in 1997 to 786 in 2011. Maputo Province showed the highest percentage reduction in MMR, while Maputo City showed a slight increase.

**Table 2:** Changes in mortality and additional lives saved for children, newborns, and mothers from 1997 to 2011

		Child (0-59 months)				Neonatal (<1 months)					Maternal			
	**Under-5 mortality rate, 1997**		**Under-5 mortality rate, 2011**	**% reduction in mortality (1997-2011)**	**Additional lives saved (1997-2011)**		**Neonatal mortality rate, 1997**	**Neonatal mortality rate, 2011**	**% reduction in mortality (1997-2011)**	**Additional lives saved (1997-2011)**	**Maternal mortality ratio, 1997**	**Maternal mortality ratio, 2011**	**% reduction in mortality (1997-2011)**	**Additional lives saved (1997-2011)**
**National**	195.62		145.43	25.7%	422 282		50.36	41.31	18.0%	85 450	870.00	785.71	9.7%	6528
**Niassa**	200.05		134.01	33.0%	18 917		52.78	40.23	23.8%	6440	870.03	693.40	20.3%	558
**Cabo Delgado**	254.52		166.71	34.5%	58 807		54.48	50.45	7.4%	2911	965.93	933.33	3.4%	181
**Nampula**	237.18		145.22	38.8%	116 474		59.61	41.14	31.0%	28 746	909.95	739.41	18.7%	1798
**Zambezia**	241.89		196.79	18.6%	92 729		64.02	54.99	14.1%	22 236	977.15	914.34	6.4%	1153
**Tete**	204.36		160.02	21.7%	22 808		51.33	42.12	17.9%	2761	876.96	757.71	13.6%	288
**Manica**	199.71		138.15	30.8%	28 812		49.52	33.91	31.5%	8138	872.40	646.82	25.9%	891
**Sofala**	210.66		143.41	31.9%	37 057		50.16	35.69	28.8%	11 077	897.40	651.44	27.4%	1316
**Inhambane**	157.54		135.78	13.8%	9009		37.20	36.38	2.2%	82	751.57	699.17	7.0%	126
**Gaza**	161.42		128.65	20.3%	15 397		32.68	33.35	-2.1%*	-248*	687.31	672.92	2.1%	1
**Maputo Provincia**	146.58		133.77	8.7%	5512		33.50	23.60	29.6%	2333	659.72	476.80	27.7%	228
**Maputo Cidade**	157.80		133.68	15.3%	16 760		30.31	30.50	-0.6%*	974*	597.12	618.53	-3.6%*	-10

*A negative mortality reduction arises when intervention coverage decreases, resulting in fewer lives saved in 2011 than in 1997.

As mentioned in the introduction, we also have estimates of mortality from the DHS surveys in 1997, 2003, and 2011, which have been used by the UN Inter-agency Group for Child Mortality Estimation (UN IGME) to create an estimated trend of U5MR over time in Mozambique. This trend shows a more significant reduction in mortality over time than our modelled estimates, with a U5MR of 209 in 1997, 148 in 2003, and 95 in 2011 [10]. This stands to reason, since LiST only accounts for indicators that are inputted into the software. It is likely that other interventions, not inputted into our projections, also had an impact on mortality, resulting in a more significant reduction than suggested by our estimates.

### Causes of death

**Table 3** shows the proportion of deaths due to different causes in 1997 and 2011. In both years, malaria was the major cause of death among children under five, with approximately one in four deaths due to malaria. There was a decrease in the proportion of deaths due to measles, and an increase in the proportion of deaths caused by AIDS and wounds. Prematurity, asphyxia, and sepsis were the major causes of neonatal death in 1997 and 2011. From 1997 to 2011 the three major causes of maternal deaths were postpartum hemorrhage, other indirect causes of death, and antepartum hemorrhage. The major causes of death among children, neonates, and mothers at provincial level followed the same pattern as the causes of death at national level.

**Table 3:** Top causes of death, in 1997 and 2011

Cause of death	1997	Cause of death	2011
**Children (0-59 months):**			
Malaria	26%	Malaria	22%
Diarrhea	12%	Other	13%
Pneumonia	12%	Pneumonia	10%
Other	10%	Diarrhea	10%
Neonatal – Prematurity	7.8%	Neonatal – Prematurity	9.0%
Neonatal – Asphyxia	7.5%	Neonatal – Asphyxia	7.3%
Neonatal – Sepsis	4.5%	AIDS	6.2%
Measles	4.2%	Neonatal – Sepsis	4.4%
Meningitis	4.0%	Wounds	4.2%
AIDS	3.0%	Meningitis	4.1%
Wounds	3.0%	Neonatal – Pneumonia	2.4%
Neonatal – Pneumonia	2.3%	Measles	2.2%
Neonatal – Other	1.4%	Neonatal – Other	1.9%
Neonatal – Congenital anomalies	1.3%	Neonatal – Congenital anomalies	1.8%
Neonatal – Tetanus	1.0%	Neonatal – Tetanus	0.7%
Whooping cough	0.7%	Whooping cough	0.6%
Neonatal – Diarrhea	0.3%	Neonatal – Diarrhea	0.3%
**Total**	**100%**	**Total**	**100%**
**Newborns (<1 months):**			
Neonatal – Prematurity	30%	Neonatal – Prematurity	32%
Neonatal – Asphyxia	29%	Neonatal – Asphyxia	26%
Neonatal – Sepsis	17%	Neonatal – Sepsis	16%
Neonatal – Pneumonia	8.7%	Neonatal – Pneumonia	8.7%
Neonatal – Other	5.3%	Neonatal – Other	6.7%
Neonatal – Congenital anomalies	5.2%	Neonatal – Congenital anomalies	6.5%
Neonatal – Tetanus	3.9%	Neonatal – Tetanus	2.5%
Neonatal – Diarrhea	1.0%	Neonatal – Diarrhea	1.1%
Total	100%	Total	100%
**Mothers:**			
Postpartum hemorrhage	24%	Other indirect	26%
Other indirect	22%	Postpartum hemorrhage	20%
Antepartum hemorrhage	13%	Antepartum hemorrhage	12%
Sepsis	11%	Sepsis	11%
Hypertensive diseases of pregnancy	11%	Hypertensive diseases of pregnancy	9.0%
Other direct	6.9%	Other direct	8.3%
Hypertensive diseases of pregnancy	4.9%	Abortion	6.1%
Abortion	4.7%	Obstructed labor	4.4%
Ectopic	1.1%	Malaria	1.6%
Malaria	1.0%	Ectopic	1.3%
**Total**	**100%**	**Total**	**100%**

### Additional lives saved

Our modelled estimates show that increases in intervention coverage from 1997 to 2011 saved an additional 422 282 child lives (0-59 months), 85 450 neonatal lives (0-1 month), and 6528 maternal lives beyond those already being saved at baseline. We calculated the number of additional lives saved for each intervention. We did this separately for the periods 1997-2011 and 2011-2015 because, as described above, we had limited data for the year 2015, and we did not want to under-represent the impact of certain interventions in the 2011-2015 period because of this lack of data. The full list of interventions and their additional lives saved is provided in Appendices S3-S5 in **Online Supplementary Document(Online Supplementary Document)**. **Tables 4 and 5** present the 10 interventions for each age category that were responsible for the most additional lives saved, resulting from the increase in coverage of these interventions beyond the baseline year (1997 for **Table 4** and 2011 for **Table 5**).

**Table 4:** Top 10 interventions for additional lives saved (1997-2015)

Intervention	National									
**Children (0-59 months):**										
	**Additional lives saved (1997-2011)**					**% of lives saved (1997-2011)**		**Additional lives saved (2011-2015)**	**% of lives saved (2011-2015)**	
Change in wasting prevalence	82 374					20%		*Data not available for 2015*		
ITN/IRS	55 757					13%		*Data not available for 2015*		
Artemisinin compounds (ACTs) for treatment of malaria	34 807					8%		11 920	41%	
Oral antibiotics for pneumonia	29 804					7%		1149	4%	
Changes in breastfeeding	27 405					6%		*Data not available for 2015*		
Measles vaccine	26 432					6%		229	1%	
Labor and delivery management	24 041					6%		3243	11%	
Change in stunting prevalence	19 257					5%		*Data not available for 2015*		
Preventing mother to child transmission (PMTCT)	19 101					5%		3621	13%	
Full supportive care, sepsis/pneumonia	15 928					4%		*Data not available for 2015*		
Other	87 376					21%		8580	30%	
**Total**	**422** **282**					**100%**		**28** **742**	**100%**	
**Newborns (<1 months):**										
Labor and delivery management		24 041			28%		3243			29%
Changes in breastfeeding		16 526			19%		*Data not available for 2015*			
Full supportive care for neonatal sepsis/pneumonia		15 928			19%		*Data not available for 2015*			
Tetanus toxoid vaccination		7867			9%		*Data not available for 2015*			
Neonatal resuscitation		6335			7%		1590			14%
Clean birth practices		3290			4%		941			8%
Thermal care		3129			4%		767			7%
Immediate assessment and stimulation		2909			3%		950			8%
Antibiotics for pPRoM		2152			3%		227			2%
Oral antibiotics for neonatal sepsis/pneumonia		1691			2%		*Data not available for 2015*			
Other		1582			2%		3572			32%
**Total**		**85** **450**			**100%**		**11** **290**			**100%**
**Mothers:**										
Labor and delivery management			3118	48%			275			31%
Active management of the third stage of labor (AMTSL)			1163	18%			135			15%
MgSO4 management of eclampsia			801	12%			93			11%
Clean birth practices			740	11%			243			27%
Antibiotics for pPRoM			462	7%			56			6%
Tetanus toxoid vaccination			97	1%			*Data not available for 2015*			
Contraceptive use			82	1%			*Data not available for 2015*			
MgSO4 management of pre-eclampsia			23	0%			2			0%
Hypertensive disorder case management			21	0%			3			0%
Malaria case management			11	0%			0			0%
Other			10	0%			77			9%
**Total**			**6528**	**100%**			**884**			**100%**

ITN/IRS – Insecticide-treated nets/indoor residual spraying, ACTs – artemisinin compounds, pPRoM – preterm premature rupture of the membranes, AMTSL – active management of the third stage of labor, MGSO_4_ – magnesium sulfate

**Table 5:** Top 10 interventions for additional lives saved (2011-2015)

Intervention	National	
	**Additional lives saved (2011-2015)**	**% of lives saved**
**Children (0-59 months):**		
Artemisinin compounds (ACTs) for treatment of malaria	11 920	41%
PMTCT	3621	13%
Labor and delivery management	3243	11%
Injectable antibiotics for neonatal sepsis/pneumonia	2977	10%
Neonatal resuscitation	1590	6%
Oral antibiotics for pneumonia	1149	4%
Immediate assessment and stimulation	950	3%
Clean birth practices	941	3%
Thermal care	767	3%
Intermittent preventive treatment of malaria during pregnancy (IPTp)	525	2%
Other	1059	4%
**Total**	**28** **742**	**100%**
**Newborns (<1 months):**		
Labor and delivery management	3243	29%
Injectable antibiotics for neonatal sepsis/pneumonia	2977	26%
Neonatal resuscitation	1590	14%
Immediate assessment and stimulation	950	8%
Clean birth practices	941	8%
Thermal care	767	7%
Intermittent preventive treatment of malaria during pregnancy (IPTp)	525	5%
Antibiotics for pPRoM	227	2%
Syphilis detection and treatment	56	0%
Preventing mother to child transmission (PMTCT)	14	0%
Other	0	0%
**Total**	**11 290**	**100%**
**Mothers:**		
Labor and delivery management	275	31%
Clean birth practices	243	27%
Active management of the third stage of labor (AMTSL)	135	15%
MgSO4 management of eclampsia	93	11%
Intermittent preventive treatment of malaria during pregnancy (IPTp)	77	9%
Antibiotics for pPRoM	56	6%
Hypertensive disorder case management	3	0%
MgSO_4_ management of pre-eclampsia	2	0%
**Other**	0	0%
**Total**	884	100%

pPRoM – preterm premature rupture of the membranes, MGSO_4_ – magnesium sulfate

For children under five between 1997 and 2011, the most additional lives saved were contributed by interventions to reduce the prevalence of wasting (82 374 or 20% of all additional lives saved), increased coverage of insecticide-treated bed nets/indoor residual spraying (55 757 or 13%), and increased coverage of ACTs for malaria (34 807 or 8%). Although we have no data for changes in wasting prevalence and ITN/IRS coverage from 2011 to 2015, increased ACT coverage contributed a high percentage (41%) of additional lives saved from 2011 to 2015, with increased coverage of prevention of mother-to-child transmission (PMTCT) contributing the second highest percentage (13%).

From 1997 to 2011, and from 2011 to 2015, increased coverage of neonatal interventions contributed 85 450 and 11 290 additional neonatal lives saved, respectively. Increased coverage of labor and delivery management (28%), breastfeeding promotion (19%), and full supportive care for neonatal sepsis/pneumonia (19%) contributed the largest proportion of additional lives saved from 1997 to 2011. From 2011 to 2015, labor and delivery management continued to add the largest proportion of lives saved (29%), with no data available for breastfeeding promotion and full supportive care for neonatal sepsis/pneumonia. Among the 6528 and 884 additional maternal lives saved for 1997-2011 and 2011-2015 respectively, the increased coverage of labor and delivery management contributed about half (58%) and one third (31%) of additional lives saved, respectively, followed by increased coverage of active management of the third stage of labor (AMTSL), magnesium-sulfate (MgSO4) for the management of eclampsia, and clean birth practices.

We have not presented our results of additional lives saved by intervention at provincial level, but these are available in Appendices S3-S5 in **Online Supplementary Document(Online Supplementary Document)**.

## DISCUSSION

Since 1997, Mozambique has seen a considerable reduction in child mortality, reflecting the efforts made by the Ministry of Health to scale up proven child health interventions and roll out new interventions. Various vaccines were introduced over the past eight years (Pentavalent3/DPT3, pneumococcal and rotavirus); a national ITN household ownership strategy was rolled out in 2011; the implementation of pediatric ART and PMTCT was introduced in 2002 in Maputo and later in the remainder of the country; and a breastfeeding promotion strategy was begun in 2009 [14]. These efforts should be recognized and celebrated for their contributions to improving child health.

Our findings also offer details about the changes in coverage over 15 years (1997-2011) that contributed the most additional lives saved among children under five – in other words, the efforts that had the biggest impact. We found that wasting and malaria interventions were responsible for most of the additional mortality reduction. However, we also found that malaria and malnutrition are still responsible for significant mortality in Mozambique, and thus continue to represent an opportunity to decrease child mortality further. In our analysis, more than one quarter of children were stunted in 2011, and malaria remains the biggest killer of children under five. A 2007 cause-of-death survey in Mozambique corroborates these findings, suggesting that malaria was responsible for 42.3% of under-five deaths [15]. These data highlight the importance and timeliness of recent further increases in government health financing to reduce under-five mortality [16].

Although increased coverage of interventions did reduce neonatal and maternal mortality in most provinces, the increase was not consistent and was limited in some areas, especially in Cabo Delgado, Gaza, and Maputo City. We found that interventions related to labor and delivery management averted more than half of the maternal deaths and more than one quarter of the neonatal deaths in the last 19 years. However, interventions related to prevention of maternal mortality such as antenatal care, skilled birth attendance, and institutional delivery showed lower coverage increases compared to other preventive and curative interventions, hence the smaller reduction in maternal mortality from 1997 to 2011 compared to child mortality. While there was an increase in coverage of facility delivery to more than 90% in Maputo Province in 2011, other provinces have yet to achieve such coverage of facility delivery or antenatal care. Additionally, only one fifth of pregnant women received preventive treatment for malaria in 2011 at national level. This may be due to a limited awareness of the consequences of malaria in pregnancy, especially for newborns [17].

The findings from this paper emphasize the success of the government in increasing coverage of existing interventions saving hundreds of thousands of additional lives, beyond those that were already being saved by existing coverage of interventions in 1997. This highlights the value of a multi-targeted approach, increasing awareness and uptake of new strategies, while further increasing access to and utilization of existing interventions. However, with all this in mind, more must be done not only to improve the coverage of interventions but also to improve the quality of such interventions, requiring more attention to policy implementation and health service delivery. Policy-makers should also ensure that gaps in intervention coverage are reduced not only in wealthy or urban areas of the country, but equitably across Mozambique. A multi-country analysis for low and middle income countries showed that both relative and absolute wealth-related and educational inequalities in maternal and neonatal health decreased in the last decade in Mozambique [18]. Nevertheless, in countries with low levels of antenatal care such as Mozambique, scaling up this service to rural and underserved areas might be an effective strategy to reduce health inequities.

### Limitations

As a retrospective analysis of secondary data, we depended on the data available to us. We were only able to include indicators modelled in LiST and indicators for which we had reliable data. Specifically, due to the limited number of interventions in the 2015 IMASIDA survey data set, we could not reliably estimate trends in mortality or causes of death for 2011-2015, or compare additional lives saved in that period with the 1997-2011 period. As such, we were only able to report most results for 1997-2011. Data limitations during the 1997-2011 period also explain why the mortality trends generated by our LiST projections (**Table 2**) under-represent the decline in mortality reported in DHS surveys from 1997, 2003, and 2011. The mortality decline in our projections only captures the coverage changes for interventions that we could model.

While these limitations raise questions about our mortality rate calculations, they do not negate the value of our intervention-specific findings on additional lives saved. Even for the 2011-2015 period, our findings reflect accurately the additional lives saved attributable to those interventions for which we have data – the lack of data on other interventions does not affect our estimates for the interventions for which we do have data. Although LiST does not allow users to produce confidence intervals for uncertain coverage estimates, this paper nonetheless offers important estimates for the relative impact of interventions, an output that can help to define priority interventions in the future. This analysis also highlights the value of using existing survey data to understand the health landscape in Mozambique, especially as routine health information system (HIS) data on mortality and causes of death are still lacking at both national and provincial levels.

## CONCLUSION

The findings from our analysis and previous surveys of mortality show that Mozambique has made great strides towards achieving MDG 4. Efforts to achieve MDG 5 have been less fruitful. Mozambique should continue striving to reduce child, neonatal, and maternal mortality – to achieve the SDGs by 2030, but more importantly to improve the lives of women and children across the country. Further commitments to interventions that we know save lives, such as ITN/IRS and improved labor and delivery management, should be sought, and additional effort should be made to monitor the impact of interventions on child and maternal health. More lives can be saved by continuing to increase coverage of existing health interventions and exploring new ways to reach underserved populations.
